# Circulating osteocrin is associated with cardiac remodeling in canine myxomatous mitral valve disease and skeletal alterations in a rat pressure-overload model

**DOI:** 10.3389/fvets.2026.1975572

**Published:** 2026-10-22

**Authors:** Masafumi Komatsu, Hiromu Udagawa, Ryuji Araki, Yuji Hamamoto, Eunryel Nam, Minoru Okamoto, Mitsuhiro Isaka

**Affiliations:** 1 Laboratory of Companion Animal Surgery, Department of Small Animal Clinical Sciences, School of Veterinary Medicine, Rakuno Gakuen University, Ebetsu, Hokkaido, Japan; 2 Yokohama Yamate Dog and Cat Medical Center, Yokohama, Kanagawa, Japan; 3 Laboratory of Clinical Pathology, Department of Small Animal Clinical Sciences, School of Veterinary Medicine, Rakuno Gakuen University, Ebetsu, Hokkaido, Japan

**Keywords:** cardiovascular biomarker, cardiovascular remodeling, heart-bone axis, MMVD, natriuretic peptides, osteocrin, pressure overload, skeletal remodeling

## Abstract

**Background:**

Myxomatous mitral valve disease (MMVD) is the most common acquired cardiac disease in dogs and is increasingly recognized as a systemic disorder associated with extracardiac remodeling. Osteocrin (OSTN), a secreted peptide implicated in natriuretic peptide biology and skeletal homeostasis, has emerged as a potential candidate biomarker of cardiovascular–skeletal interactions. However, circulating OSTN dynamics during cardiovascular disease progression remain incompletely characterized.

**Methods:**

We employed a translational study design integrating a clinical cohort of dogs with naturally occurring myxomatous mitral valve disease (MMVD) and an experimental rat pressure-overload remodeling model. Circulating OSTN concentrations were evaluated alongside cardiac biomarkers, echocardiographic indices, bone mineral density, skeletal mechanical properties, and tissue localization by immunohistochemistry.

**Results:**

In the canine cohort (*n* = 47), circulating OSTN concentrations demonstrated stage-dependent alterations and were significantly associated with echocardiographic indices of cardiac remodeling. In the rat pressure-overload model, circulating OSTN concentrations increased, whereas NT-proBNP did not show a significant pressure-overload-associated increase. In pooled analyses, circulating OSTN was inversely associated with bone mineral density and stiffness; however, these associations were substantially influenced by between-group separation, whereas no significant associations were observed between NT-proBNP and the skeletal parameters examined. Multivariable regression analysis demonstrated that ACVIM disease stage remained significantly associated with circulating OSTN concentrations after adjustment for age and body weight.

**Conclusion:**

Circulating OSTN was associated with cardiac remodeling in dogs with MMVD and with skeletal alterations in an experimental rat pressure-overload model. These findings support further investigation of OSTN as a candidate biomarker at the interface between cardiovascular and skeletal remodeling.

## 1 Introduction

Myxomatous mitral valve disease (MMVD) is the most common acquired cardiac disease in dogs and is characterized by progressive valvular degeneration, mitral regurgitation, cardiac remodeling, and, in advanced disease, congestive heart failure. Disease severity is clinically classified according to the American College of Veterinary Internal Medicine (ACVIM) staging system (1). Heart failure and chronic cardiovascular disease are increasingly recognized as systemic disorders extending beyond impaired cardiac performance to involve multiple organ systems (2–5). Skeletal abnormalities, including reduced bone mineral density and increased fracture risk, have also been reported in patients with cardiovascular disease and heart failure (6–8).

Natriuretic peptide signaling represents a biologically plausible pathway linking cardiovascular and skeletal physiology (9–13). Natriuretic peptide receptor-C (NPR-C) regulates natriuretic peptide clearance and influences tissue-specific peptide availability (14–16). However, endogenous regulators of natriuretic peptide clearance during cardiovascular disease remain incompletely understood.

Osteocrin (OSTN), also described as musclin, is a secreted peptide expressed in bone and skeletal muscle and is involved in the regulation of natriuretic peptide clearance through NPR-C interactions (17–20). Beyond its established role in skeletal biology, OSTN has been detected in cardiac tissues and has been implicated in cardiovascular stress responses and cardioprotection in experimental models (21–23). Importantly, whether circulating OSTN concentrations are associated with disease severity in naturally occurring cardiovascular disease and with skeletal remodeling across complementary translational models remains unresolved.

Therefore, we hypothesized that circulating OSTN is associated with cardiovascular remodeling and skeletal alterations across complementary clinical and experimental models. To test this hypothesis, we evaluated circulating OSTN concentrations and their associations with cardiac remodeling in a clinical canine MMVD cohort and with skeletal alterations in a complementary experimental pressure-overload rat model.

## 2 Materials and methods

### 2.1 Study design

This study employed a translational study design integrating a clinical cohort of dogs with naturally occurring myxomatous mitral valve disease (MMVD) and an experimental rat pressure-overload remodeling model. The study aimed to evaluate associations between circulating osteocrin (OSTN), natriuretic peptide-related biomarkers, cardiac remodeling indices, and skeletal parameters across clinical and experimental cardiovascular disease settings.

### 2.2 Clinical study: canine MMVD cohort

From April 2019 to May 2020, 47 client-owned dogs presented to the Yokohama Yamate Dog and Cat Medical Center or the Rakuno Gakuen University Veterinary Teaching Hospital were enrolled in the study. Dogs diagnosed with MMVD and clinically healthy controls were included. Dogs with other primary cardiac diseases were excluded.

Comorbidities secondary to MMVD were permitted. All owners provided informed consent prior to enrollment. Physical examination, medical history, and medication use information were recorded. Thoracic radiography and transthoracic echocardiography were performed as part of the clinical evaluation, with individual parameters analyzed when available. Because some echocardiographic measurements were unavailable, analyses involving echocardiographic variables were performed using available cases for each parameter, and the corresponding sample size is reported for each analysis. MMVD severity was classified according to the American College of Veterinary Internal Medicine (ACVIM) consensus guidelines (1) into stage B1, B2, and C/D. Dogs without structural or functional cardiac abnormalities served as healthy controls. Echocardiographic examinations were performed using standard imaging protocols by experienced operators using commercially available ultrasound systems. Parameters assessed included left atrial–to–aorta ratio (LA/Ao), left ventricular internal diameters in diastole and systole (LVIDd and LVIDs), fractional shortening (FS), left ventricular ejection fraction (LVEF). Vertebral heart size (VHS) was measured from thoracic radiographs. Information regarding age, body weight, medication exposure, and clinical history was collected to evaluate potential confounding factors associated with circulating biomarker concentrations.

### 2.3 Experimental study: pressure-overload rat model

Six-week-old female Slc:Wistar rats obtained from Japan SLC were assigned to pressure-overload (*n* = 6) or control (*n* = 6) groups. Female rats were selected because osteoporosis is more prevalent in women than in men, and the experimental design was therefore focused on female animals in the context of cardiovascular–skeletal interactions (24). General anesthesia was induced with 4%−5% sevoflurane and maintained with 2%−3% sevoflurane. Pressure overload was induced by abdominal aortic constriction (AAC), an established rat model of pressure-overload cardiac remodeling (25). Briefly, the abdominal aorta was constricted immediately distal to the renal artery branch using 6-0 nylon to achieve a diameter of approximately 0.7 mm. Control animals underwent sham surgery involving the same surgical procedures, including laparotomy, but without aortic constriction. Body temperature was maintained using a heating pad throughout surgery. Buprenorphine (0.05 mg/kg, subcutaneously) was administered preoperatively for analgesia. Postoperative pain was assessed based on general clinical condition, including activity and appetite, and additional buprenorphine (0.05 mg/kg, subcutaneously) was administered as needed. Animals were followed for 21 days to evaluate biomarker dynamics and skeletal functional alterations associated with pressure-overload remodeling. At the experimental endpoint, animals were deeply anesthetized with sevoflurane and euthanized by intravenous administration of potassium chloride (100–150 mg/kg). The experimental model was intended to represent an early pressure-overload remodeling phenotype rather than a comprehensively characterized heart failure model. Direct hemodynamic measurements, including the trans-stenotic pressure gradient and left ventricular pressure, were not performed in the present study. Blood samples for biomarker measurements were collected before surgery and at 21 days after surgery.

### 2.4 Biomarker analysis

Serum OSTN concentrations were measured in both dogs and rats, whereas serum NT-proBNP concentrations were measured in rats, using commercially available enzyme-linked immunosorbent assay (ELISA) kits. Serum samples were analyzed in duplicate, and biomarker concentrations were calculated from standard curves generated for each assay. Detailed assay characteristics and assay-specific conditions, including manufacturer and catalog information, assay format, detection range, analytical sensitivity, intra-assay and inter-assay coefficients of variation, and sample dilution conditions, are summarized in Supplementary Table S1.

### 2.5 Bone mineral density and biomechanical testing

Bone mineral density (BMD) of the entire right femur, including both cortical and trabecular bone, was assessed using an iNSiGHT dual-energy X-ray absorptiometry system (OsteoSys). BMD was determined from the projected area of the entire femur following X-ray irradiation. Mechanical properties of the right femur, including maximum load and stiffness, were evaluated using a three-point bending test with MZ-500S/MZ-500D testing systems (Maruto), using a support span of 13 mm and a loading speed of 2 mm/min.

### 2.6 Histological assessment of cardiac remodeling

Cardiac tissues were fixed, paraffin-embedded, sectioned, and stained with hematoxylin and eosin (H&E) for histological assessment. Left ventricular wall thickness (LVWT) and interventricular septal (IVS) thickness were quantitatively assessed in cardiac sections from animals for which appropriate histological sections were available (control, *n* = 5; pressure-overload, *n* = 4). Group data are presented as mean ± SD. Comparisons between the control and pressure-overload groups were performed using Welch's two-tailed unpaired *t*-test, with *P* < 0.05 considered statistically significant.

### 2.7 Immunohistochemistry

Cardiac tissues were fixed in 4% paraformaldehyde, paraffin-embedded, and sectioned at a thickness of 4 μm. Antigen retrieval was performed using Tris-EDTA antigen retrieval solution (pH 9.0; Code 415201, Nichirei Biosciences, Tokyo, Japan) at 121 °C for 10 min. Sections were incubated with a rabbit anti-OSTN polyclonal antibody (bs-9566R; Bioss, Woburn, MA, USA) at a dilution of 1:100, followed by detection using a polymer-based detection system (Histofine Simple Stain Mouse MAX-PO(R), Code 414341; Nichirei Biosciences Inc., Tokyo, Japan). Immunoreactivity was visualized using 3,3′-diaminobenzidine tetrahydrochloride (DAB; Kanto Chemical Co., Inc., Tokyo, Japan) as the chromogen. Negative controls were performed using normal rabbit serum in place of the primary antibody or by omitting the primary antibody, followed by the same secondary antibody detection procedure.

### 2.8 Statistical analysis

Statistical analyses were performed using R software (version 4.2.0). The normality of continuous variables was assessed using the Shapiro–Wilk test. Continuous variables among the four canine groups were compared using one-way analysis of variance followed by the Tukey–Kramer test for normally distributed data, or the Kruskal–Wallis test followed by appropriate *post hoc* multiple comparisons for non-normally distributed data. Categorical variables were compared using the chi-square test. Specifically, serum OSTN concentrations among the four canine groups (Figure 1) were compared using the Kruskal–Wallis test followed by Dunn's *post hoc* test for multiple comparisons. Correlations between serum OSTN concentrations and cardiac parameters in dogs (Figure 2) were assessed using Spearman's rank correlation coefficient. Normality was evaluated using the Shapiro–Wilk test; because several cardiac variables did not satisfy the normality assumption, Spearman's rank correlation was used consistently for the canine correlation analyses. In the rat pressure-overload model, changes in circulating OSTN and NT-proBNP concentrations over time (Figure 3) were analyzed using two-way repeated-measures analysis of variance to evaluate the effects of group, time, and the group × time interaction, followed by Sidak's multiple-comparisons test for *post hoc* comparisons. Bone mineral density and biomechanical parameters, including maximum load and stiffness, were compared between the control and pressure-overload groups (Figure 4) using unpaired Student's *t*-tests. Associations between circulating biomarkers (OSTN and NT-proBNP) and skeletal parameters, including bone mineral density, maximum load, and stiffness (Figure 5), were assessed using Spearman's rank correlation analysis. In addition to the pooled analyses including all animals, within-group correlations were examined separately in control and pressure-overload animals to assess the potential influence of between-group separation on the pooled associations. Because the within-group sample size was small (*n* = 6 per group), these subgroup analyses were considered exploratory. Given the exploratory nature of the study, no global adjustment for multiple testing was applied across the secondary correlation analyses. Accordingly, *P* values from these analyses should be interpreted as exploratory. Multiple-comparison adjustments were applied where specified for prespecified *post hoc* comparisons. Statistical significance was defined as *P* < 0.05.

**Figure 1:**
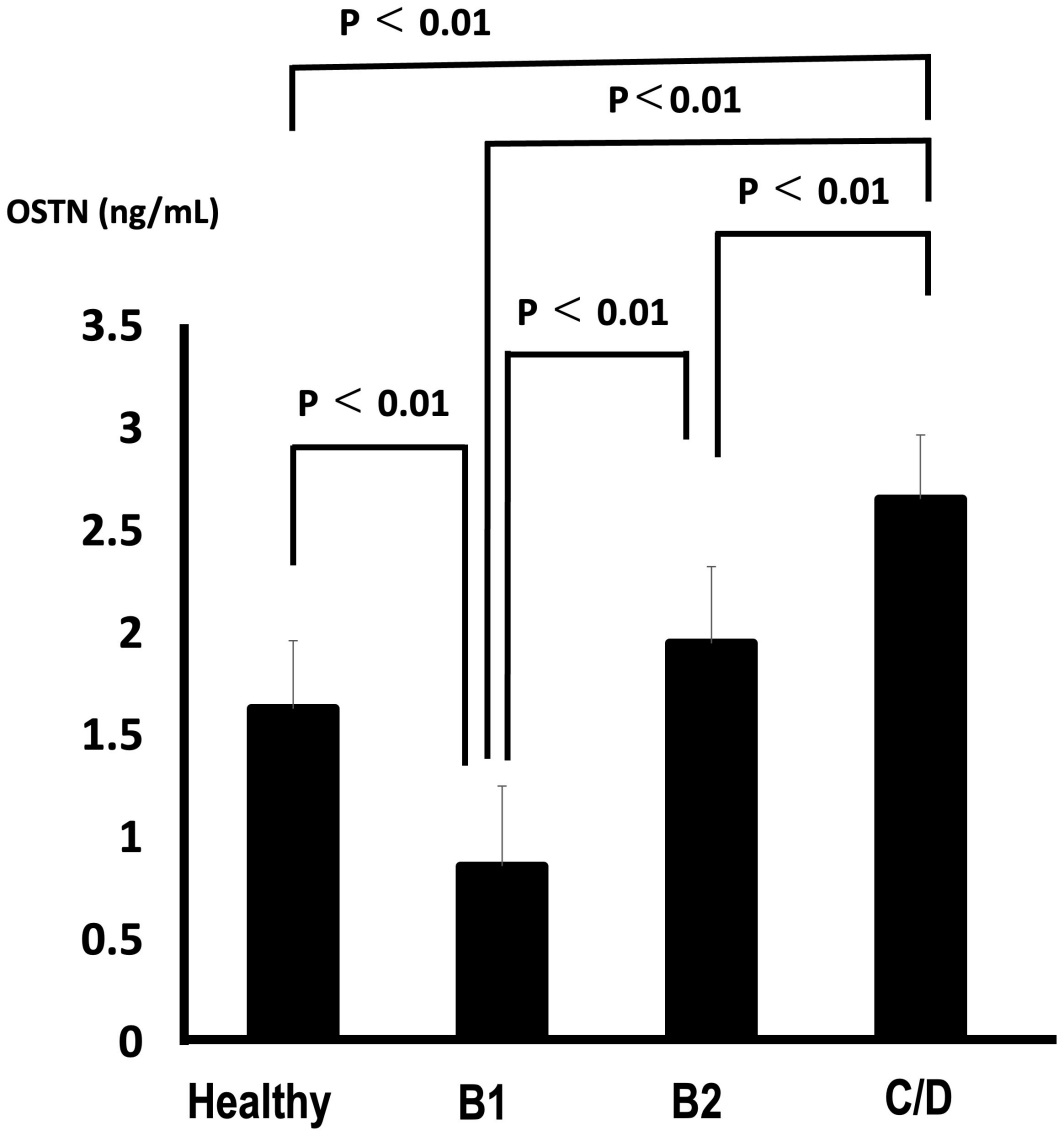
Serum osteocrin (OSTN) concentrations across disease stages in dogs with myxomatous mitral valve disease (MMVD). Serum OSTN concentrations were evaluated in healthy dogs (*n* = 16) and dogs with MMVD classified according to the American College of Veterinary Internal Medicine guidelines as stage B1 (*n* = 10), B2 (*n* = 14), and C/D (*n* = 7). Serum OSTN concentrations were significantly lower in stage B1 than in healthy dogs and dogs with stage B2 MMVD, whereas concentrations in stage C/D were significantly higher than those in healthy dogs and dogs with stage B1 and B2 MMVD. No significant difference was observed between healthy dogs and dogs with stage B2 MMVD. Data are presented as mean ± SD. Differences among groups were assessed using the Kruskal–Wallis test followed by Dunn's *post hoc* test for multiple comparisons. Statistical significance is indicated in the figure.

**Figure 2:**
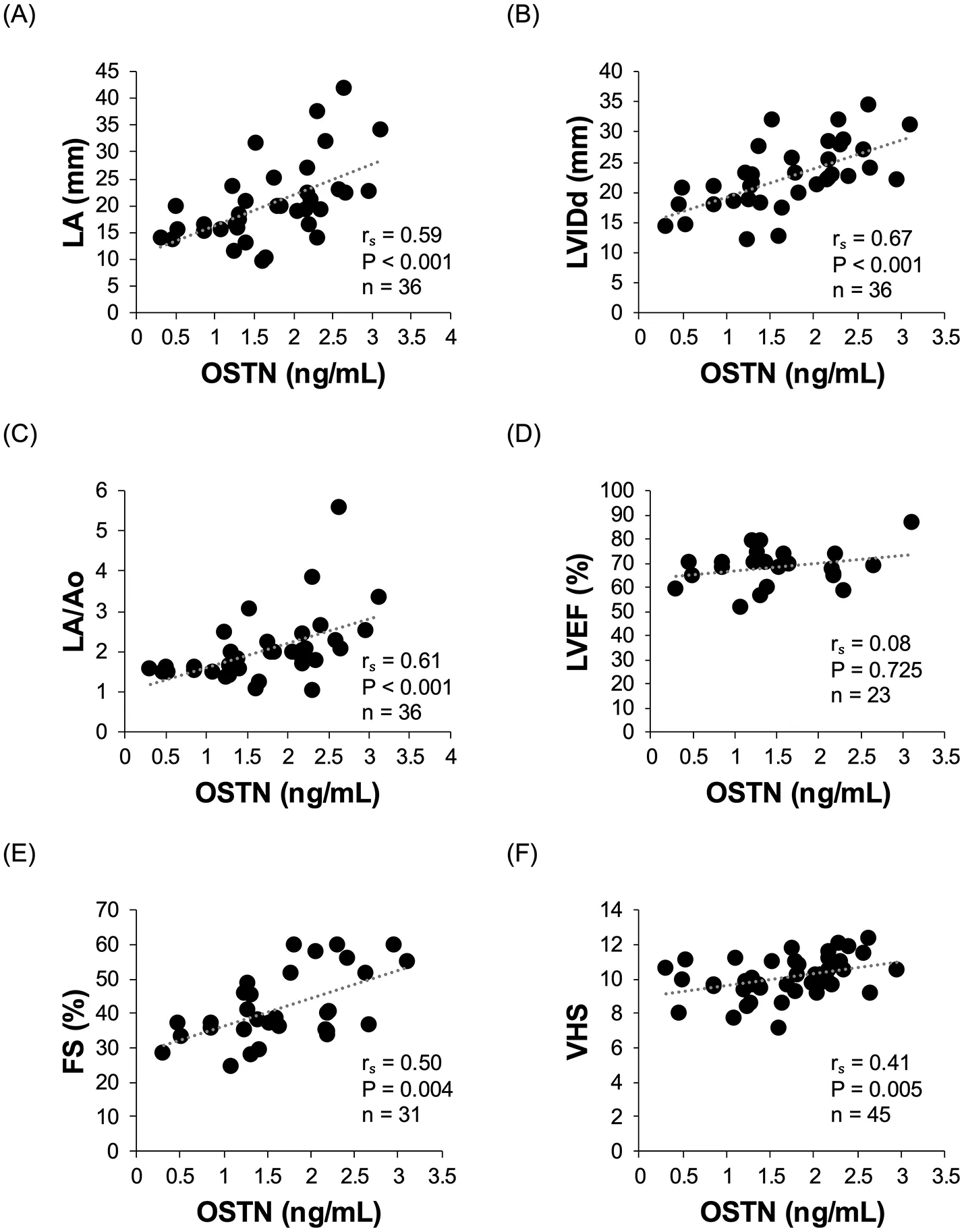
Correlations between circulating osteocrin (OSTN) concentrations and cardiac remodeling indices in dogs with myxomatous mitral valve disease (MMVD). Scatter plots show the relationships between serum OSTN concentrations and **(A)** left atrial dimension (LA; *n* = 36), **(B)** left ventricular internal diameter in diastole (LVIDd; *n* = 36), **(C)** left atrial-to-aortic ratio (LA/Ao; *n* = 36), **(D)** left ventricular ejection fraction (LVEF; *n* = 23), **(E)** fractional shortening (FS; *n* = 31), and **(F)** vertebral heart size (VHS; *n* = 45). Correlations were assessed using Spearman's rank correlation coefficient. Spearman's rank correlation coefficients (*r*_*s*_) and corresponding *P* values are shown in each panel.

**Figure 3:**
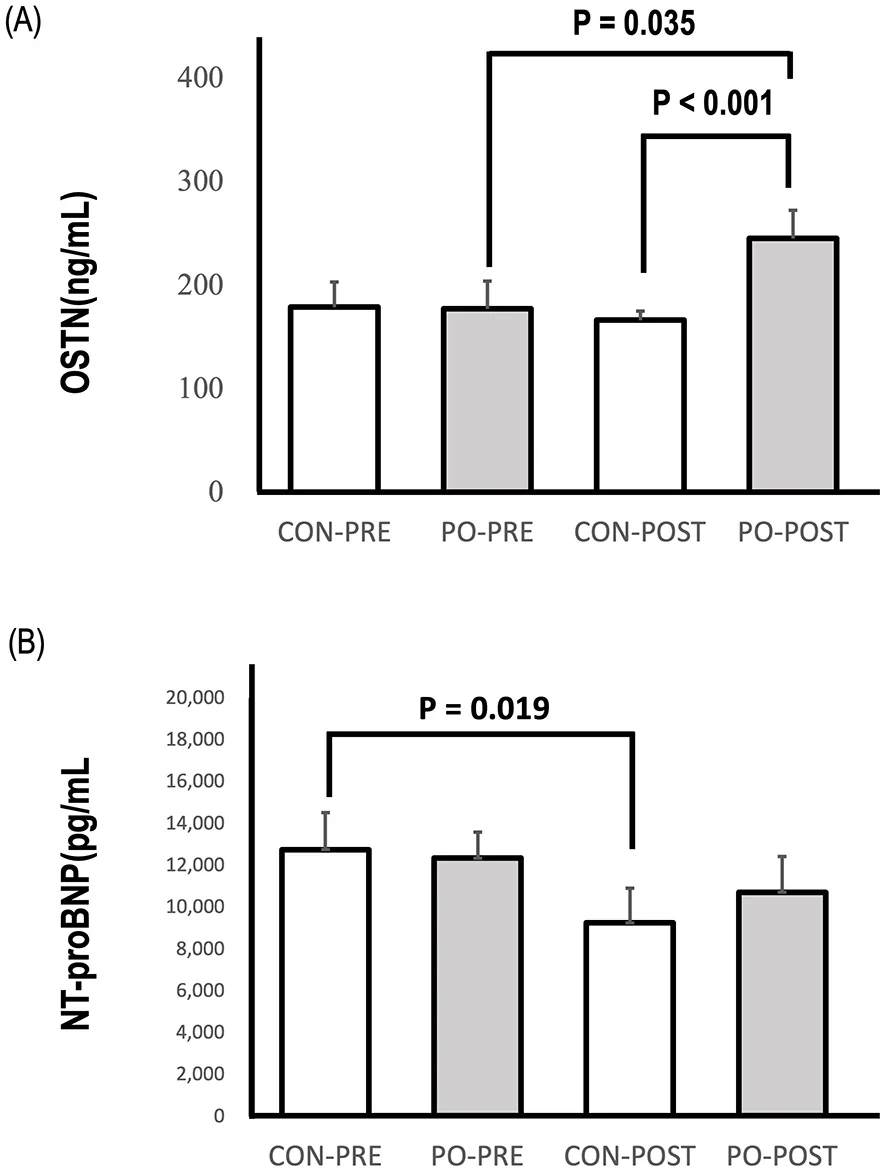
**(A, B)** Changes in circulating N-terminal pro-B-type natriuretic peptide (NT-proBNP) and osteocrin (OSTN) concentrations in the pressure-overload rat remodeling model. Serum NT-proBNP and OSTN concentrations were measured before (pre) and three weeks after (post) abdominal aortic constriction or sham surgery in control (*n* = 6) and pressure-overload (*n* = 6) groups. Data are presented as mean ± SD. Changes over time were analyzed using two-way repeated-measures ANOVA to evaluate the effects of group, time, and the group × time interaction, followed by Sidak's multiple-comparisons test for *post hoc* comparisons. Statistical significance is indicated in the figure.

**Figure 4:**
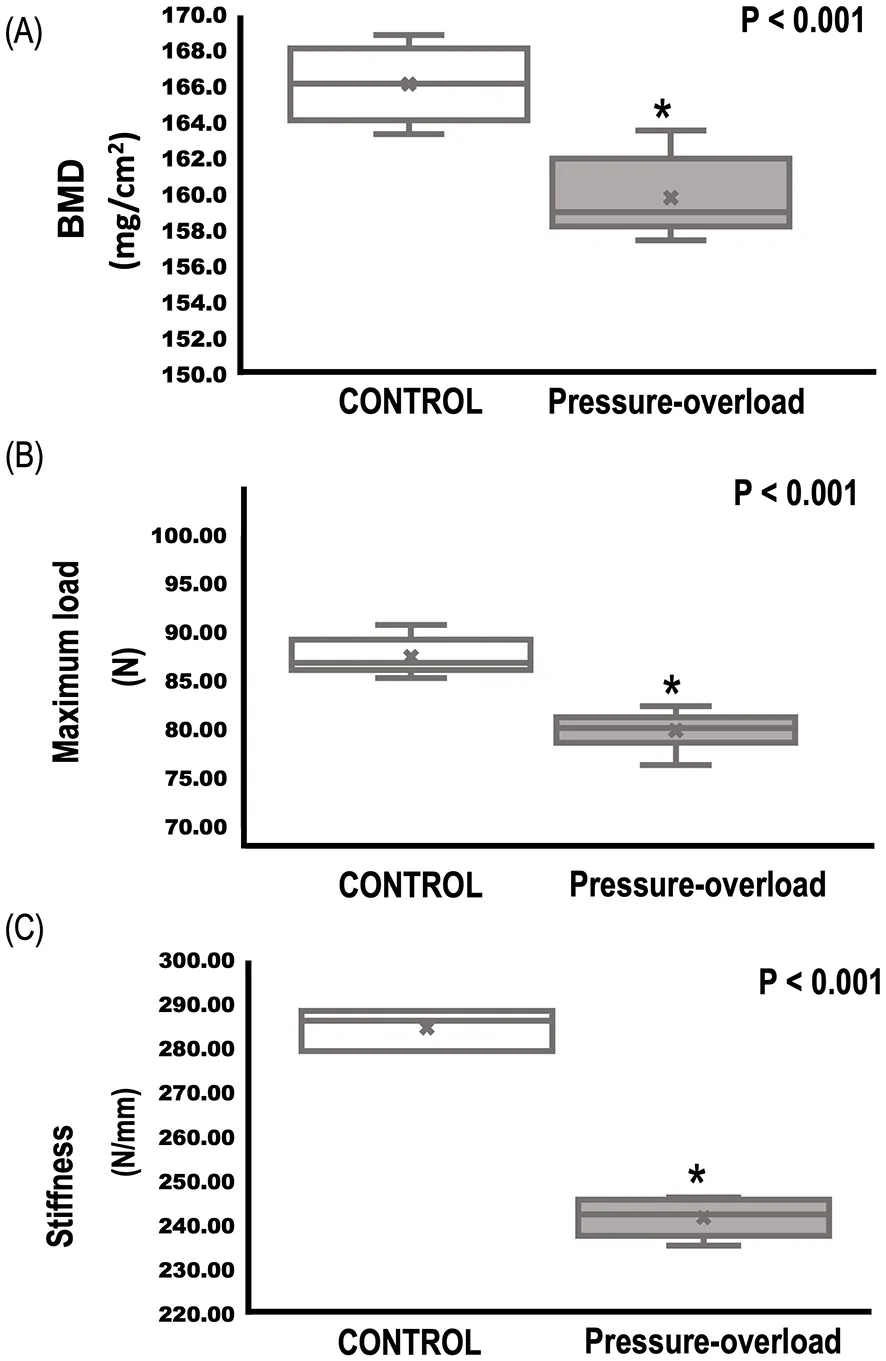
**(A–C)** Bone mineral density (BMD) and biomechanical properties in the pressure-overload rat remodeling model. BMD was assessed by dual-energy X-ray absorptiometry (DXA), and femoral biomechanical properties were evaluated by maximum load and stiffness in control (*n* = 6) and pressure-overload (*n* = 6) groups. Data are presented as mean ± SD. Comparisons between the control and pressure-overload groups were performed using unpaired Student's t-tests. Statistical significance is indicated in each panel.

**Figure 5:**
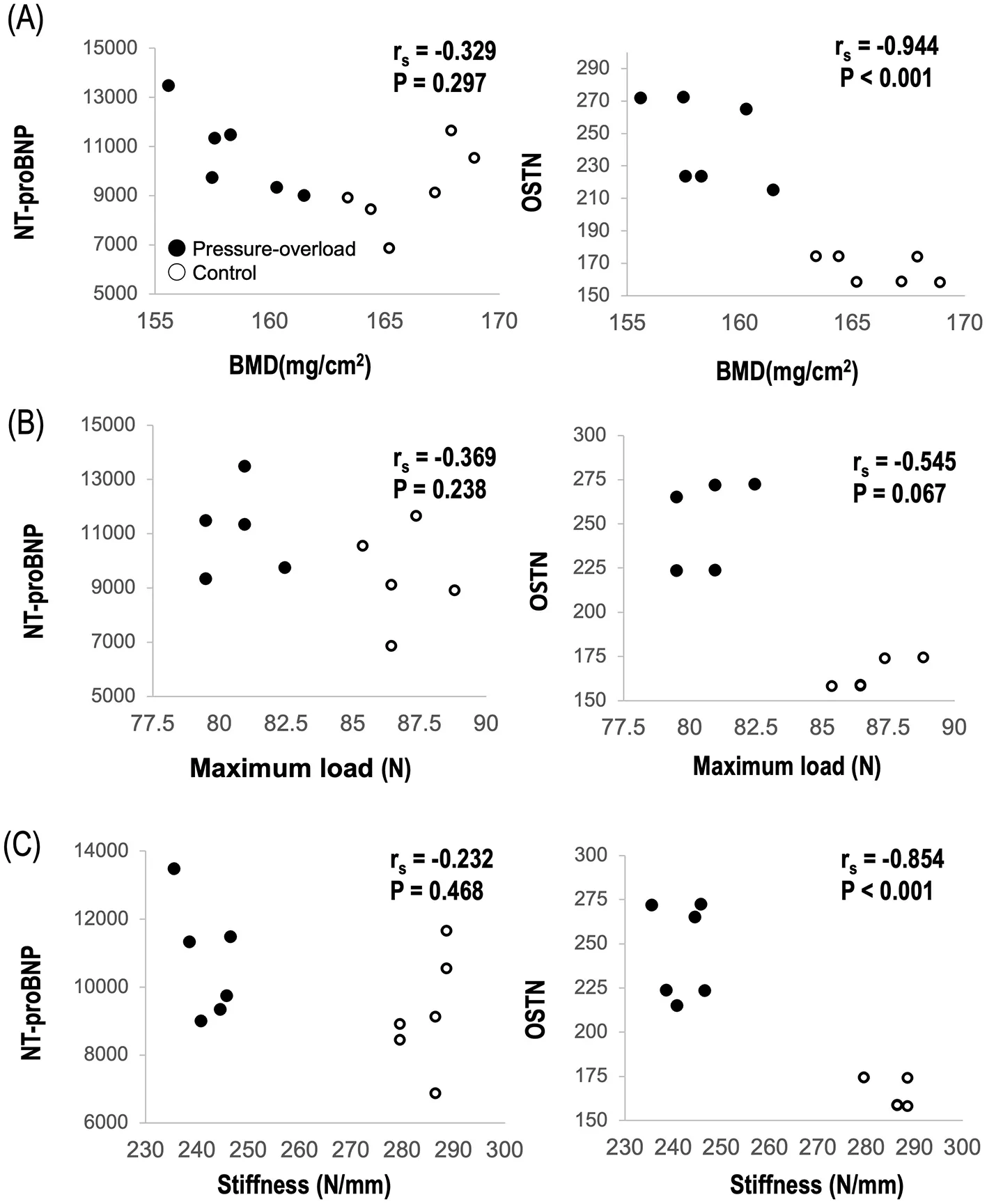
Associations between skeletal parameters and circulating biomarkers in the pressure-overload rat remodeling model. Scatter plots show the associations of circulating NT-proBNP (left panels) and OSTN (right panels) with **(A)** bone mineral density, **(B)** maximum load, and **(C)** stiffness. Control and pressure-overload animals are shown separately using open and filled symbols, respectively. Spearman's rank correlation coefficients (*r*_*s*_) and corresponding *P* values were calculated for the pooled dataset (*n* = 12). For the associations between OSTN and bone mineral density and between OSTN and stiffness, additional exploratory within-group correlations were calculated separately for control and pressure-overload animals (*n* = 6 per group) to assess the potential influence of between-group separation. These strong pooled associations were not statistically significant within either experimental group.

### 2.9 Multivariable analysis

Multivariable linear regression analysis was performed using circulating serum OSTN concentration as the dependent variable. ACVIM disease stage, age, and body weight were included as explanatory variables. Healthy dogs were used as the reference category for ACVIM disease stage. Only dogs with complete data for all variables were included in the multivariable analysis. One clinically healthy dog had missing age and body weight data and was therefore excluded from the multivariable regression analyses, resulting in a complete-case sample of 46 dogs. Model assumptions were assessed by evaluation of residual normality, homoscedasticity, and multicollinearity. Because breed distribution differed among ACVIM groups, with Chihuahuas overrepresented in the MMVD groups, a sensitivity analysis was additionally performed by including breed (Chihuahua vs. other breeds) as an additional covariate in the multivariable model. Breed was dichotomized as Chihuahua vs. other breeds because of the small numbers of individual non-Chihuahua breeds.

## 3 Results

### 3.1 Clinical characteristics of the canine cohort

A total of 47 dogs were enrolled in this study, including 31 dogs diagnosed with MMVD and 16 clinically healthy controls. Based on ACVIM classification, dogs with MMVD were categorized as stage B1 (*n* = 10), B2 (*n* = 14), and C/D (*n* = 7). There were no significant differences in age, sex distribution, body weight, or body condition score among the groups, whereas breed distribution differed significantly among the groups, with Chihuahuas being more frequently represented in the MMVD groups than in the healthy group (*P* = 0.013). LA/Ao, LVIDd, FS, and VHS differed significantly among the groups, with generally higher values in more advanced disease stages, whereas LVIDs and LVEF did not differ significantly among the groups. ACE inhibitor, pimobendan, and loop diuretic use differed significantly among the groups and was more frequent in dogs with advanced MMVD, whereas spironolactone use did not differ significantly among the groups. Detailed clinical and echocardiographic characteristics are summarized in Table 1.

**Table 1:** Baseline clinical characteristics of healthy dogs and dogs with myxomatous mitral valve disease (MMVD).

Variable	Healthy (*n* = 16)	B1 (*n* = 10)	B2 (*n* = 14)	C/D (*n* = 7)	*P*-value	Statistical test
Number of dogs, n	16	10	14	7	–	Descriptive
Age (years)	9.1 ± 4.0 (*n* = 15)	11.0 ± 1.8	11.9 ± 2.3	11.7 ± 1.4	0.203	Kruskal–Wallis
Sex, male/female	11/5	7/3	5/9	3/4	0.199	Chi-square
Breed, Chihuahua/other	6/10	9/1	11/3	6/1	0.013	Chi-square
Body weight (kg)	4.9 ± 3.4 (*n* = 15)	3.1 ± 1.3	3.1 ± 1.1	3.1 ± 1.3	0.215	Kruskal–Wallis
BCS	3.1 ± 0.6 (*n* = 15)	3.0 ± 0.5	3.1 ± 0.9	2.9 ± 0.4	0.905	Kruskal–Wallis
LA/Ao	1.4 ± 0.2 (*n* = 5)	1.6 ± 0.1 (*n* = 9)	2.0 ± 0.5 (*n* = 10)	3.1 ± 1.3 (*n* = 6)	<0.001	Kruskal–Wallis
LVIDd (mm)	16.7 ± 4.4 (*n* = 5)	19.2 ± 3.8	24.9 ± 3.9	27.1 ± 4.6	<0.001	one-way ANOVA
LVIDs (mm)	10.6 ± 4.2 (n = 5)	12.9 ± 2.0 (n = 9)	14.2 ± 2.3 (n = 13)	14.3 ± 3.4 (n = 6)	0.152	Kruskal-Wallis
FS (%)	33.6 ± 4.5 (n = 5)	36.1 ± 7.0 (n = 9)	46.2 ± 9.9 (n = 11)	49.0 ± 10.6 (n = 6)	0.006	one-way ANOVA
LVEF (%)	66.2 ± 7.4 (*n* = 5)	66.3 ± 7.5 (*n* = 8)	70.4 ± 7.7 (*n* = 7)	73.9 ± 11.3 (*n* = 3)	0.868	Kruskal-Wallis
VHS	9.5 ± 1.0 (*n* = 15)	9.6 ± 1.0	10.6 ± 0.8	11.2 ± 1.2 (*n* = 6)	<0.001	one-way ANOVA
ACE inhibitor use, *n* (%)	1 (6.2%)	1 (10.0%)	3 (21.4%)	4 (57.1%)	0.032	Chi-square
Pimobendan use, *n* (%)	0 (0.0%)	1 (10.0%)	9 (64.3%)	5 (71.4%)	<0.001	Chi-square
Loop diuretic use, *n* (%)	0 (0.0%)	0 (0.0%)	1 (7.1%)	4 (57.1%)	<0.001	Chi-square
Spironolactone use, *n* (%)	0 (0.0%)	0 (0.0%)	1 (7.1%)	1 (14.3%)	0.366	Chi-square

Data are presented as mean ± SD or *n* (%), as appropriate. Values in parentheses indicate the number of dogs with available data when data were missing. Group comparisons were performed using one-way ANOVA, the Kruskal–Wallis test, or the chi-square test, as indicated. BCS, body condition score; LA/Ao, left atrial-to-aortic root ratio; LVIDd, left ventricular internal diameter in diastole; LVIDs, left ventricular internal diameter in systole; FS, fractional shortening; LVEF, left ventricular ejection fraction; VHS, vertebral heart score. Breed categories were summarized as Chihuahua versus other breeds because of sparse counts across individual breeds.

### 3.2 Serum osteocrin concentrations

Serum OSTN concentrations differed significantly among the study groups (Figure 1). *Post hoc* comparisons demonstrated that dogs with stage B1 MMVD had significantly lower serum OSTN concentrations than healthy controls and dogs with stage B2 MMVD. In contrast, dogs with stage C/D MMVD had significantly higher serum OSTN concentrations than healthy controls and dogs with stage B1 and B2 MMVD. Serum OSTN concentrations did not differ significantly between healthy controls and dogs with stage B2 MMVD. These findings demonstrate stage-dependent alterations in circulating OSTN concentrations across MMVD severity groups.

### 3.3 Correlations between serum OSTN concentrations and cardiac parameters

Serum OSTN concentrations were significantly positively correlated with LA (*r*_*s*_ = 0.59, *P* < 0.001, *n* = 36), LVIDd (*r*_*s*_ = 0.67, *P* < 0.001, *n* = 36), LA/Ao (*r*_*s*_ = 0.61, *P* < 0.001, *n* = 36), FS (*r*_*s*_ = 0.50, *P* = 0.004, *n* = 31), and VHS (*r*_*s*_ = 0.41, *P* = 0.005, *n* = 45). In contrast, no significant correlation was observed between serum OSTN concentration and LVEF (*r*_*s*_ = 0.08, *P* = 0.725, *n* = 23) (Figure 2).

### 3.4 Multivariable regression analysis

Multivariable linear regression analysis was performed in 46 dogs with complete data, using serum OSTN concentration as the dependent variable and ACVIM disease stage, age, and body weight as explanatory variables. After adjustment for age and body weight, ACVIM disease stage remained significantly associated with circulating OSTN concentrations. Compared with healthy dogs, dogs with stage B1 MMVD had significantly lower circulating OSTN concentrations (β = −0.759, 95% CI −1.074 to −0.443, *P* < 0.001), whereas dogs with stage B2 (β = 0.321, 95% CI 0.020–0.622, *P* = 0.037) and stage C/D MMVD (β = 1.027, 95% CI 0.671–1.383, *P* < 0.001) had significantly higher circulating OSTN concentrations. Neither age nor body weight was significantly associated with circulating OSTN concentrations. The multivariable model explained 74.0% of the variability in serum OSTN concentrations (*R*^2^ = 0.740; adjusted *R*^2^ = 0.708; overall *P* < 0.001) (Table 2). Because Chihuahuas were disproportionately represented across ACVIM groups, a sensitivity analysis was performed with breed (Chihuahua vs. other breeds) included as an additional covariate. Chihuahua breed was not independently associated with circulating OSTN concentration (β = 0.050, 95% CI −0.263 to 0.363, *P* = 0.750). After additional adjustment for breed, the associations for stage B1 (β = −0.784, *P* < 0.001) and stage C/D (β = 1.002, *P* < 0.001) remained statistically significant, whereas the association for stage B2 was attenuated and no longer reached statistical significance (β = 0.299, *P* = 0.080). The sensitivity model included 46 dogs and had an *R*^2^ of 0.741 (adjusted *R*^2^ = 0.701; overall *P* < 0.001) (Supplementary Table S2).

**Table 2:** Multivariable linear regression analysis of factors associated with circulating OSTN concentrations in dogs.

Variable	β coefficient	SE	95% CI	*P*-value
ACVIM stage B1 vs. healthy	−0.759	0.156	−1.074 to −0.443	<0.001
ACVIM stage B2 vs. healthy	0.321	0.149	0.020 to 0.622	0.037
ACVIM stage C/D vs. healthy	1.027	0.176	0.671 to 1.383	<0.001
Age, per year	0.011	0.020	−0.028 to 0.051	0.56
Body weight, per kg	0.031	0.025	−0.020 to 0.082	0.229

The dependent variable was serum OSTN concentration (ng/mL). Clinically healthy dogs were the reference category. β coefficients are unstandardized regression coefficients and represent adjusted mean differences in serum OSTN concentration. The multivariable model included ACVIM stage, age, and body weight. Complete-case analysis included 46 dogs. Model fit: *R*^2^ = 0.740; adjusted *R*^2^ = 0.708; overall *P* < 0.001. CI, confidence interval; SE, standard error; OSTN, osteocrin; ACVIM, American College of Veterinary Internal Medicine.

### 3.5 Pressure-overload rat model

To further investigate the relationship between circulating biomarkers and cardiovascular–skeletal remodeling in a complementary experimental model, serum biomarkers and skeletal parameters were evaluated in rats subjected to abdominal aortic constriction (AAC). For circulating OSTN, two-way repeated-measures ANOVA demonstrated significant effects of group (*P* = 0.0018) and time (*P* = 0.016), as well as a significant group × time interaction (*P* = 0.0018). Sidak-adjusted *post hoc* comparisons showed a significant increase in OSTN concentrations from pre- to post-intervention in the pressure-overload group (*P* = 0.035) and significantly higher post-intervention OSTN concentrations in the pressure-overload group than in controls (*P* < 0.001). For NT-proBNP, a significant effect of time was observed (*P* < 0.001), whereas neither the group effect (*P* = 0.493) nor the group × time interaction (*P* = 0.117) was significant. Sidak-adjusted *post hoc* analysis showed a significant decrease from pre- to post-intervention in the control group (*P* = 0.019); however, no significant pressure-overload-specific NT-proBNP response was detected. Dual-energy X-ray absorptiometry demonstrated significantly reduced bone mineral density in the pressure-overload group compared with controls (*P* < 0.001). Furthermore, biomechanical testing revealed significant reductions in maximum load (*P* < 0.001) and stiffness (*P* < 0.001), indicating impaired skeletal biomechanical properties. Collectively, these findings demonstrate that pressure overload was associated with increased circulating OSTN concentrations and impaired skeletal properties, without a corresponding significant pressure-overload-specific increase in NT-proBNP (Figures 3, 4).

### 3.6 Histological assessment of cardiac remodeling

Left ventricular wall thickness (LVWT) was greater in pressure-overload rats than in controls (2,281.3 ± 137.5 vs. 1,879.3 ± 263.2 μm, respectively; *P* = 0.0245). Interventricular septal (IVS) thickness was also significantly greater in the pressure-overload group than in controls (2,625.6 ± 300.0 vs. 2,084.4 ± 300.5 μm, respectively; *P* = 0.0331) (Figure 6).

**Figure 6:**
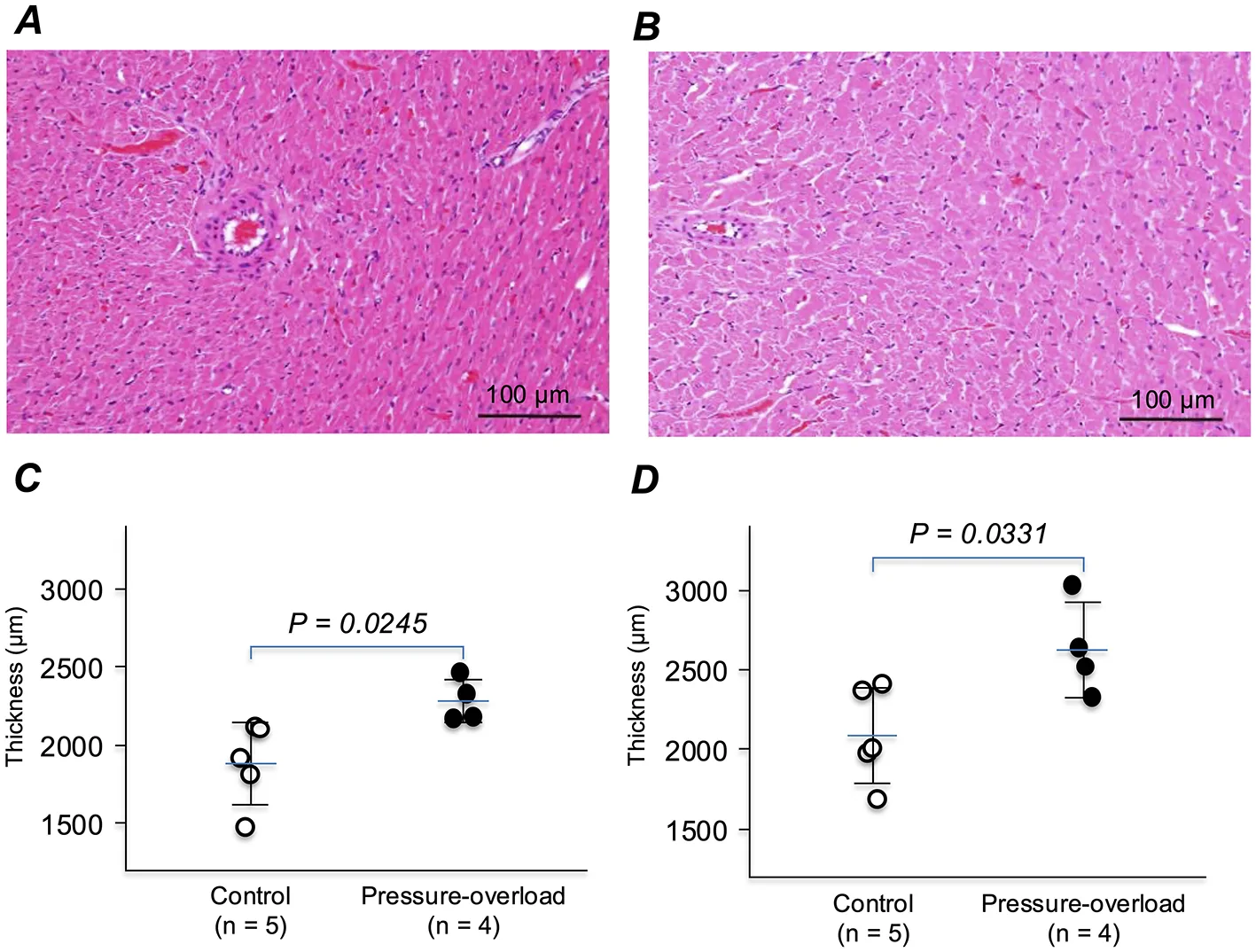
Histological assessment of cardiac remodeling in the pressure-overload rat model. Representative hematoxylin and eosin (H&E)-stained myocardial sections from **(A)** control and **(B)** pressure-overload rats at the same magnification (×20 objective; scale bars = 100 μm). Quantitative assessment of **(C)** left ventricular wall thickness (LVWT) and **(D)** interventricular septal (IVS) thickness. Both LVWT and IVS thickness were significantly greater in the pressure-overload group than in the control group. Individual data points represent individual animals, and data are presented as mean ± SD (control, *n* = 5; pressure-overload, *n* = 4). Comparisons were performed using Welch's two-tailed unpaired *t*-test. LVWT, *P* = 0.0245; IVS thickness, *P* = 0.0331.

### 3.7 Correlations between circulating biomarkers and skeletal parameters

In the pooled analysis of control and pressure-overload animals (*n* = 12), serum OSTN concentrations were inversely correlated with bone mineral density (*r*_*s*_ = −0.944, *P* < 0.001) and stiffness (*r*_*s*_ = −0.854, *P* < 0.001), whereas the inverse association with maximum load did not reach statistical significance (*r*_*s*_ = −0.545, *P* = 0.067). Because the experimental groups showed substantial separation in both circulating OSTN concentrations and skeletal parameters, additional within-group analyses were performed. The association between OSTN and bone mineral density did not reach statistical significance within either the control group (*r*_*s*_ = −0.771, *P* = 0.072) or the pressure-overload group (*r*_*s*_ = −0.771, *P* = 0.072). Similarly, the association between OSTN and stiffness was not statistically significant within the control group (*r*_*s*_ = −0.717, *P* = 0.109) or the pressure-overload group (*r*_*s*_ = −0.086, *P* = 0.872). In the pooled analysis, NT-proBNP concentrations were not significantly associated with bone mineral density (*r*_*s*_ = −0.329, *P* = 0.297), maximum load (*r*_*s*_ = −0.369, *P* = 0.238), or stiffness (*r*_*s*_ = −0.232, *P* = 0.468). These findings indicate that the strong pooled associations between OSTN and bone mineral density or stiffness were substantially influenced by between-group separation and should therefore be interpreted cautiously (Figure 5).

### 3.8 Immunohistochemical localization of OSTN in cardiac tissue

Immunohistochemical analysis demonstrated OSTN immunoreactivity predominantly in cells lining the atrioventricular valvular surface. In contrast, myocardial fibers showed no detectable OSTN immunoreactivity under the conditions examined. Representative images from control and pressure-overload rats are shown in Figure 7.

**Figure 7:**
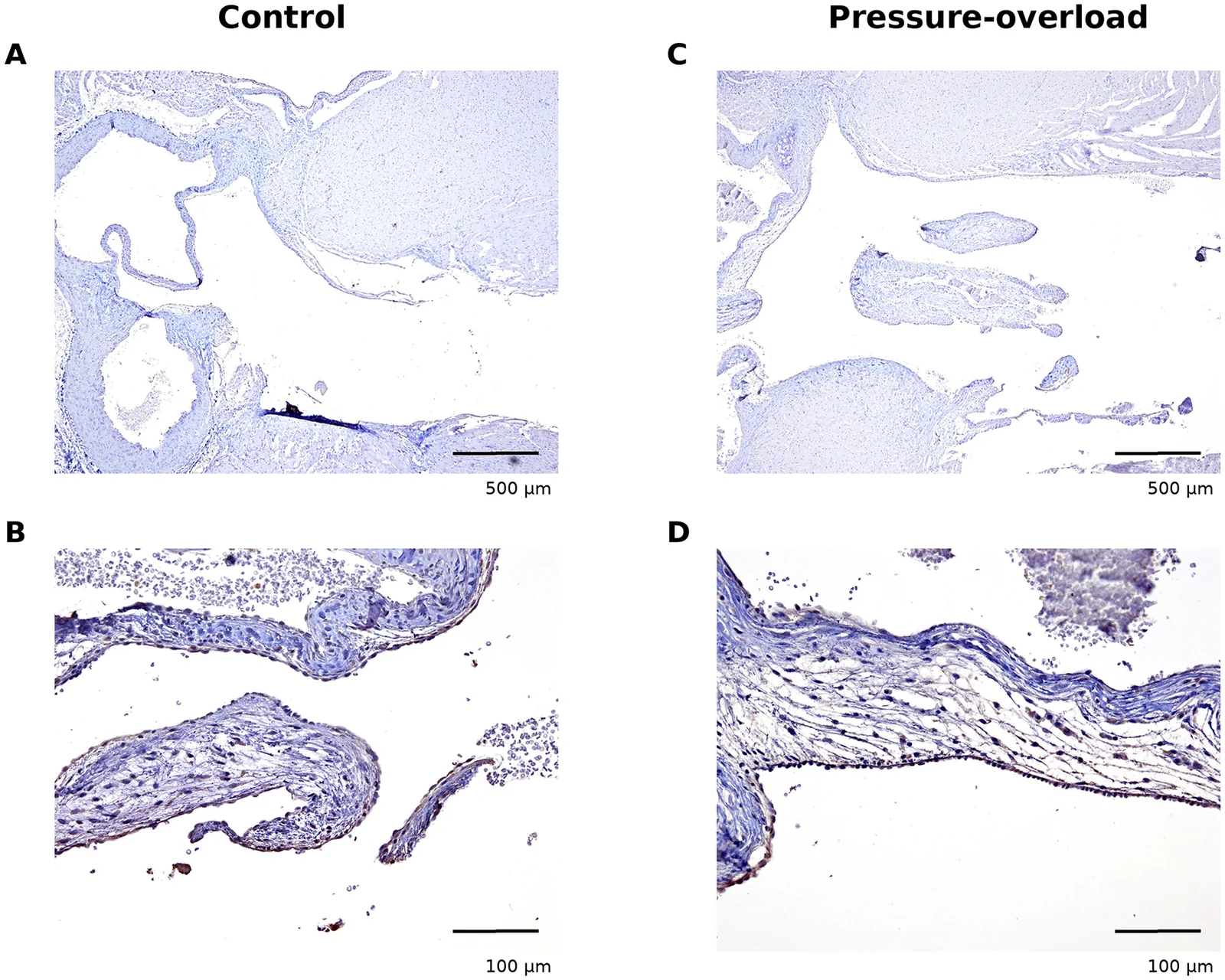
Immunohistochemical localization of osteocrin (OSTN) in atrioventricular valve tissue. Representative low- and high-magnification immunohistochemical images from control **(A, B)** and pressure-overload **(C, D)** rats. Low-magnification images **(A, C)** show the atrioventricular valvular region, whereas higher-magnification images **(B, D)** demonstrate OSTN immunoreactivity in cells lining the valvular surface. The low- and high-magnification images are representative images and are not serial magnifications of the same microscopic field. Scale bars = 500 μm **(A, C)** and 100 μm **(B, D)**.

## 4 Discussion

The present findings support the concept that cardiovascular remodeling may be accompanied by alterations beyond the cardiovascular system, including changes in skeletal properties. Bone-derived endocrine factors are increasingly recognized as potential mediators of cardiovascular physiology and disease (26). Within this framework, the present study investigated circulating OSTN as a candidate biomarker associated with cardiovascular and skeletal remodeling.

In dogs with naturally occurring MMVD, circulating OSTN concentrations exhibited stage-dependent alterations and were associated with echocardiographic indices of cardiac remodeling. These observations suggest that circulating OSTN concentrations are associated with disease stage and indices of cardiac remodeling in dogs with MMVD. Although breed distribution differed among disease groups, sensitivity analysis additionally adjusting for Chihuahua breed showed that breed itself was not independently associated with circulating OSTN, and the associations for stages B1 and C/D remained significant. However, the association for stage B2 was attenuated after breed adjustment, indicating that residual breed-related confounding cannot be completely excluded. Experimental studies have implicated OSTN in cardiovascular stress responses and have reported cardioprotective effects in models of cardiac injury and cardiomyopathy (20–23). The lower circulating OSTN concentrations observed in stage B1 remain unexplained and may reflect biological heterogeneity or residual confounding related to clinical characteristics not fully captured in the present analysis. Given the exploratory nature and limited sample size of the present cohort, this observation requires validation in larger longitudinal studies. Importantly, the present observational findings should not be interpreted as evidence of causality or direct endocrine regulation.

Abdominal aortic constriction is an established experimental approach for inducing chronic left ventricular pressure-overload remodeling (25). In the present study, histological assessment demonstrated significantly increased left ventricular wall thickness and interventricular septal thickness in AAC-treated rats, supporting the presence of structural cardiac remodeling. However, trans-stenotic pressure gradients and left ventricular pressures were not directly measured. Therefore, the magnitude of the hemodynamic load imposed by AAC cannot be quantified from the present data, and the histological findings should be interpreted as evidence of structural remodeling rather than direct confirmation of the degree of left ventricular pressure overload or established heart failure. Accordingly, we conservatively refer to the experimental condition as a pressure-overload remodeling model. In this experimental pressure-overload remodeling model, circulating OSTN concentrations increased in association with alterations in skeletal parameters, whereas NT-proBNP did not show a significant pressure-overload-associated increase. NT-proBNP is a well-established biomarker widely used in the clinical assessment of heart failure (27); however, a significant increase was not detected in the present 21-day pressure-overload model. The absence of a significant NT-proBNP response may reflect the relatively early remodeling stage represented by the 21-day experimental period. However, because cardiac function and hemodynamic load were not comprehensively characterized, the physiological basis for the lack of a significant NT-proBNP increase cannot be determined from the present data. Notably, circulating OSTN changed despite the absence of a significant pressure-overload-associated increase in NT-proBNP, indicating different biomarker response patterns in the present experimental model. Whether OSTN responds earlier than NT-proBNP during cardiovascular remodeling cannot be determined from the present study and requires longitudinal investigation with detailed cardiac phenotyping.

In the experimental model, pressure-overloaded rats exhibited significant reductions in DXA-derived bone mineral density, maximum load, and stiffness, demonstrating concurrent alterations in bone density and skeletal biomechanical properties. These findings indicate that both bone mineral density and biomechanical properties were altered in association with experimental pressure overload. In the pooled analysis, circulating OSTN showed strong inverse associations with bone mineral density and stiffness, whereas NT-proBNP was not significantly associated with the skeletal parameters examined. However, additional analyses performed separately within the control and pressure-overload groups did not demonstrate statistically significant associations between OSTN and either bone mineral density or stiffness. Thus, the strong pooled associations were substantially influenced by separation between the experimental groups and should not be interpreted as evidence of a continuous dose-response relationship between circulating OSTN concentrations and skeletal deterioration at the individual-animal level. Nevertheless, the coordinated group-level increase in circulating OSTN and reductions in skeletal parameters are consistent with concurrent cardiovascular and skeletal remodeling following pressure overload.

Immunohistochemical analysis localized OSTN immunoreactivity predominantly to cells lining the atrioventricular valvular surface in both experimental groups, whereas myocardial fibers showed no detectable immunoreactivity under the conditions examined. Cardiomyocyte-associated OSTN expression has been reported during zebrafish cardiac development; however, evidence defining the cellular localization of OSTN within mammalian cardiac tissue remains limited (21). The present localization of OSTN immunoreactivity to cells lining the valvular surface suggests that non-myocyte cardiac structures may represent a cardiac compartment associated with OSTN expression. Differences in species, developmental stage, and cardiac structure may account, at least in part, for the apparent differences between previous observations and the present findings. Nevertheless, immunohistochemical localization alone cannot establish the cellular source of circulating OSTN or determine whether valvular OSTN expression is altered by pressure overload. Further studies incorporating quantitative tissue expression analyses and cell-specific approaches are required to determine whether valvular OSTN contributes to circulating OSTN concentrations and to clarify its potential role in cardiovascular remodeling.

Several limitations should be acknowledged. First, the observational design precludes causal inference regarding the relationship between circulating OSTN concentrations and cardiovascular or skeletal remodeling. Second, mechanistic interrogation of the OSTN–NPR-C–natriuretic peptide axis, including downstream cGMP signaling pathways, bone turnover markers, and skeletal cellular mechanisms, was not performed. Third, the experimental pressure-overload model was intended to represent an early remodeling phenotype rather than a comprehensively characterized heart failure model. Although increased left ventricular wall and interventricular septal thickness provided histological evidence of structural remodeling, direct hemodynamic measurements, including trans-stenotic pressure gradients and left ventricular pressures, were not performed. Therefore, the magnitude of pressure overload could not be quantified, and the experimental phenotype should not be interpreted as a comprehensively characterized heart failure model. In addition, only female rats were included in the experimental study. Because sex-dependent differences in cardiovascular and skeletal responses were not examined, the present findings cannot be directly generalized to male animals. An additional limitation concerns the correlation analyses in the rat model. The strong pooled correlations between OSTN and skeletal parameters were substantially influenced by separation between the control and pressure-overload groups. Within-group analyses did not reach statistical significance, although these exploratory analyses were limited by the small sample size (*n* = 6 per group). Accordingly, the present data do not establish a continuous within-group relationship between circulating OSTN concentrations and the severity of skeletal alterations. In the canine cohort, conventional cardiac biomarkers such as NT-proBNP were not measured. Therefore, direct comparison between circulating OSTN and established cardiac biomarkers could not be performed in dogs, limiting assessment of the incremental biomarker value of OSTN in canine MMVD. Future studies incorporating established cardiac biomarkers are needed to determine whether OSTN provides complementary information beyond conventional markers of cardiac disease. Furthermore, although breed-adjusted sensitivity analysis did not identify Chihuahua breed as an independent predictor of circulating OSTN, the limited representation of non-Chihuahua breeds restricts definitive assessment of breed-specific effects. In addition, because no global adjustment for multiple testing was applied across the secondary correlation analyses, these findings should be considered exploratory and interpreted cautiously. Echocardiographic data were incomplete for several variables, and missing measurements were more frequent among clinically healthy dogs. Accordingly, analyses of associations between circulating OSTN and echocardiographic parameters were based on available cases, and potential selection bias related to non-random missingness cannot be excluded. Finally, the cross-sectional clinical design, limited experimental sample size, and biomarker assessment at only two time points constrain inference regarding temporal biomarker trajectories, including whether changes in circulating OSTN precede those in NT-proBNP, as well as longitudinal disease progression and causal relationships. In addition, CKD was documented as a clinical comorbidity in 7 of 47 dogs; however, complete renal biochemical data, including BUN and serum creatinine concentrations, were not available across the cohort. Therefore, dogs could not be consistently classified according to azotemia status, and renal function variables were not included in the multivariable regression model. Residual confounding related to renal function therefore cannot be excluded.

Taken together, the present study demonstrates that circulating OSTN concentrations are associated with cardiac remodeling in naturally occurring canine MMVD and with skeletal alterations in an experimental pressure-overload model. These findings support further investigation of OSTN as a candidate biomarker associated with cardiovascular–skeletal remodeling and provide a translational framework for studying heart–bone interactions. These observations are consistent with emerging concepts of bone–vascular cross-talk and bone as an endocrine organ (26, 28, 29). Future longitudinal and mechanistic studies will be required to determine whether circulating OSTN directly participates in cardiovascular–skeletal communication or primarily serves as a biomarker reflecting coordinated tissue remodeling.

## Data Availability

The raw data supporting the conclusions of this article will be made available by the authors, without undue reservation.
