# Supplementary material for: Circulating osteocrin is associated with cardiac remodeling in canine myxomatous mitral valve disease and skeletal alterations in a rat pressure-overload model
Source: Front Vet Sci. 2026 Oct 22;13:1975572. doi: 10.3389/fvets.2026.1975572 (PMC13735096; doi:10.3389/fvets.2026.1975572)
Supplement: Supplementary file 1 [file Data_Sheet_1.pdf]

Supplementary Table S1. Characteristics of the ELISA kits used for biomarker measurements.

Biomarker	Species	Manufacturer	Catalog No.	Assay format	Sample type	Detection range	Sensitivity (LOD)	Intra-assay CV	Inter-assay CV	Sample dilution	Duplicate measurement
OSTN	Canine	MyBioSource	MBS752942	Competitive ELISA	Serum	0–25 ng/mL	0.1 ng/mL	<10%	<10%	According to the manufacturer's protocol	Samples analyzed in duplicate
OSTN	Rat	MyBioSource	MBS017565	Sandwich ELISA	Serum	0.25–8 ng/mL	0.1 ng/mL	<15%	<15%	5-fold	Samples analyzed in duplicate
NT-proBNP	Rat	MyBioSource	MBS2881463	Sandwich ELISA	Serum	15.6–1000 pg/mL	<7.86 pg/mL	≤4.7%	≤6.9%	According to the manufacturer's protocol	Samples analyzed in duplicate

Abbreviations: CV, coefficient of variation; LOD, limit of detection; NT-proBNP, N-terminal pro-B-type natriuretic peptide; OSTN, osteocrin.
