# Supplementary material for: Circulating osteocrin is associated with cardiac remodeling in canine myxomatous mitral valve disease and skeletal alterations in a rat pressure-overload model
Source: Front Vet Sci. 2026 Oct 22;13:1975572. doi: 10.3389/fvets.2026.1975572 (PMC13735096; doi:10.3389/fvets.2026.1975572)
Supplement: Supplementary file 2 [file Data_Sheet_2.pdf]

Supplementary Table S2. Sensitivity analysis of factors associated with circulating OSTN concentrations in dogs after additional adjustment for breed.

Variable	β coefficient	SE	95% CI	P-value
ACVIM stage B1 vs healthy	−0.784	0.176	−1.141 to −0.427	< 0.001
ACVIM stage B2 vs healthy	0.299	0.166	−0.037 to 0.634	0.08
ACVIM stage C/D vs healthy	1.002	0.195	0.608 to 1.396	< 0.001
Age, per year	0.015	0.022	−0.030 to 0.060	0.511
Body weight, per kg	0.035	0.028	−0.022 to 0.091	0.223
Chihuahua vs other breeds	0.050	0.155	−0.263 to 0.363	0.75

The dependent variable was serum OSTN concentration (ng/mL). Clinically healthy dogs and non-Chihuahua breeds were used as the reference categories. β coefficients are unstandardized regression coefficients and represent adjusted mean differences in serum OSTN concentration. This sensitivity model included ACVIM stage, age, body weight, and breed (Chihuahua vs. other breeds). Complete-case analysis included 46 dogs. Model fit: $R^2 = 0.741$; adjusted $R^2 = 0.701$; overall $P < 0.001$. CI, confidence interval; SE, standard error; OSTN, osteocrin; ACVIM, American College of Veterinary Internal Medicine.
